# Diseases of the Maxillary Sinus

**Published:** 1901-06-15

**Authors:** Thos. L. Gilmer

**Affiliations:** Chicago, Ills.


THE
DENTAL REGISTER.

Vol. LV. June 15, 1901. No. 6.

### DISEASES OF THE MAXILLARY SINUS.

BY THOS. L. GILMER, M.D., D.D.S., CHICAGO, ILLS.

Read at the Southwestern Dental Association, April 10, 1901.

Diseases of the antrum of Highmore are of frequent occurrence. This is especially true since the epidemic, which has of late years prevailed, known as la grippe, as they are a frequent sequelæ of that disease. Diseases of this cavity either yield easily to treatment or they are stubborn. The diseases to which the antrum are subject are: Empyema, suppuration, mucous engorgement, and also to polypi, cysts and other pathological growths. A simple engorgement, from an increase of the secretion from the lining membrane, with stenosis or diminution in size, or a closure of the osteum maxillaire is usually, although the symptoms may be very marked and painful, easily cured by evacuation of the fluid. Empyema or suppurations, however, are not in some cases so easily cured. Some of the symptoms of disease of this cavity are: Pain in the cheek; bulging of the eye by pressure on the orbital plate by the contents of the cavity; bulging of the cheek with absorption of the bone from the same cause, or there may be a flow of pus into the nose, or through a sinus into the mouth. Most of these symptoms may appear at the same time, and in addition, there may be amaurosis or blindness in the eye on the affected side from pressure on the optic nerve.

Suppuration is the most common disease of the antrum, and in some instances it is very difficult to cure, requiring months or even years of treatment for a cure.

It does not necessarily follow because there is pus in the antrum that it originates in that cavity. Formerly we have supposed that the secretion from the frontal sinuses and the ethmoid cells was always discharged into the middle meatus of the nose through the infundibulum, but more recent research by Cryer and others has discovered that it is not uncommon for the fluids from these two sources to find exit by the infundibulum directly into the maxillary sinus. In case of disease of the frontal sinus, in which pus is formed, and the opening of the infundibulum such as to connect directly with the antrum, pus will be emptied into this cavity and the antrum may be treated indefinitely without the least diminution of the pus or a relief of the condition.

The natural opening of the antrum is between the middle and inferior terbinated bones. This opening is not at the most dependent portion of the sinus, and on this account drainage can not be complete ; therefore, if from any cause a greater amount of fluid is excreted than is necessary to moisten the walls of the cavity, it does not readily escape and may become the cause of a purulent excretion by its becoming infected with pyogenic microbes. It would seem that the antrum was badly formed, having its natural outlet about half way up from the base of the cavity ; but I suppose when we were created we were not supposed to become diseased. I am of the opinion that catarrhal inflammations are the most common causes of engorgements and suppurations of the antrum of Highmore. It is not unreasonable to suppose that the sinuses of the head may, and do, participate in catarrhal inflammations which so often affect the nasal mucous membranes, as the lining of this cavity is continuous with the maxillary, frontal and the other adjoining cavities. A corizal inflammation generally does affect all of the tissues in immediate continuity. When an individual is under the influence of an inflammatory condition of the Schneiderian membrane, there is often pain in the cheek which indicates inflammatory conditions in the maxillary sinus ; he also has pain in the region of the frontal sinus, which indicates that the inflammation has invaded that cavity also. In many, the osteum maxillaire is naturally small, and, in case of swelling by catarrhal inflammation of the mucous membrane covering the turbinated bones, this opening may be closed firmly. If the inflammation becomes chronic there may be thickening of the indurated tissue, which may permanently close the opening. In that case, if there be but a slight increase of the normal secretions, it may cause a serious condition by putrefactive changes which may take place if the fluid becomes infected. In that case noxious gases are formed, and there being no outlet, pain and inflammatory action is set up, which increases the flow of the normal fluid. This may fill the cavity and produce engorgement or perhaps suppuration, as the conditions offer the best opportunity for the growth and multiplication of pyogenic and other organisms.

The teeth may cause diseases of the antrum. If a number of skulls be examined, in some of them it will be found that there are, corresponding to the buccal roots, small elevations on the buccal side of the floor of the antrum. If the bone be removed from the summit of one of these protuberances it will be found that it is very thin, and in other instances the bone over the apex of the roots nearly or quite penetrated the floor of the cavity. It is readily understood that if the pulps die in these roots and septic matter remains in them an inflammation may spring up which will extend to the floor of the sinus and the whole cavity may become diseased by it. On the other hand, in cases where the roots nearly penetrate the cavity, if there be an inflammation of its lining membrane, the pulps in the teeth may be destroyed by the inflammation extending from the lining membrane to them, which in some instances may account for the death of pulps in sound molars.

The treatment of engorgements is perfect drainage and antiseptic irrigation. By some it is recommended that an opening be made into the sinus through an enlargement of the natural opening, if closed, re-establishing it by means of a trocar. To me this is a difficult operation, and the fact that when it is opened it may not remain patulous, or, if it does, the most dependent portion of the sinus is not drained, necessitating the patient lying on one side to evacuate the cavity, besides the difficulty of irrigating it, causes me to believe that it is never the best practice to enter from this direction. The old objection that an opening made through the bone on the buccal side of the jaw will not close is simply not generally true; indeed, it is often difficult to keep it open sufficiently long for the purposes of treatment. If a first molar is the cause of the trouble, or if it is too badly decayed to be again made serviceable, it may be extracted and an opening secured at the most dependent portion of the cavity by entering the antrum through the anterior buccal root socket. If this tooth be too valuable to be removed, and the second bicuspid or second molar be of no value, then either of these may be removed and the sinus entered from the socket. But the location of the first molar offers by far the best point of entrance, if the cavity is to be entered through a tooth's socket. In the majority of cases I prefer to open into the maxillary sinus just above and buccally to the anterior buccal root of the first molar, because we here enter the cavity at its most dependent portion, and, the bone being thin, we readily gain ample access, and the opening for the treating of empyema or the removal of polypi, should be sufficiently large to permit the introduction of the little finger for exploration. If there be a simple engorgement by fluids and gases, the pain is relieved as soon as the cavity is emptied, and this may be all that is necessary to cause a cure, but in such cases the cavity should be irrigated with the normal salt solution once or twice a day for a few days. If, however, there be suppuration, then further treatment will be necessary. It may be that irrigation, first with the salt solution, then with pyrozone as a detergent, followed by a few drops of the oil of cassia in a glass of water, will suffice, but in many cases there are conditions present which need further treatment. In some instances the cavity is divided by bony partitions, and these may have to be cut away before the seat of the disease can be reached. In some instances dead bone is found as a cause of the trouble. If so, this, of course, must be removed, or nature given an opportunity to cast it off. In other cases we find the tissues covering the walls of the cavity in such a condition that they must first be curetted before the parts can be made to take on healthy action. Unless the parts yield readily to treatment the cavity opening should be increased in size so that the sinus may be explored with the little finger, as the tactile sense of the finger is much more reliable for examination than are probes. I do not use plugs to close the opening made in the antrum, and rarely employ tubes. If the opening be made through the bone from the buccal side, instead of directly upward, as through the socket of a tooth, the cheek folding over the opening prevents particles of food entering and the drainage which is so desirable is secured, while if it be plugged up we defeat a part of the intention in making the opening. If tubes are employed they should be only sufficiently long to reach the bottom of the sinus, if they extend above this point drainage is not possible until the secretion fills up the cavity above the end of the tube.

I said that in some cases there was a condition of the lining membrane of the antrum which would not yield to treatment until the surface was thoroughly curetted. In these cases we find a thickening of the mucous membrane with purulent discharges, and in some instances ulcers here and there. This condition may have been brought about by long-continued bathing of the lining membrane by acrid secretions or pus discharges from adjacent cavities or tissues; by catarrhal secretions from the nose, or pus from the apex of the roots of teeth. Antiseptic medication alone in these cases, even if the primary cause be removed, does not always cure the condition. By curetting the surface, removing unhealthy granulations, then antiseptic medication may lead the way to a growth of new and healthy tissue, and with the aid of stimulant antiseptic medication, the parts will probably be returned to a normal condition. For irrigating purposes, a weak salt solution is an excellent cleansing antiseptic and stimulating wash. If there is pus, 3 percent pyrozone may be employed. In addition to this, I would recommend for your consideration as a detergent or cleansing wash a solution of Carl Siler's antiseptic tablets. Parenthetically, I may say, that while many persons may not have a serious catarrhal condition of the nose, some do have accumulations in the nose which may remain there sufficiently long to become fetid, rendering the breath offensive. You are aware of the fact that you come in very close contact with your patients, and if your breath has an odor caused by the above condition, I would recommend that you have in your operating case a bottle of these tablets, and also spraying apparatus, and that you spray out the nasal cavities with a solution of the tablets, and by this means keep the nose in a wholesome condition.

Oil of cassia is stimulating and antiseptic. It may be added to a saturated solution of boracic acid, one drop to two ounces of the solution. Sulphate of zinc, two grains to the ounce, is also stimulating and astringent, and excellent in an irrigant. A weak solution of iodin is much thought of by some in the treatment of diseases of the lining membrane of the antrum. If there is odor, permanganate of potash solution is an excellent deodorant irrigant.

In opening the antrum as indicated a burring engine with drills and burrs may be employed, or a chisel, etc. Some employ the trocar with a guard to prevent the point of the instrument going too far into the sinus and injuring the walls of the cavity. (I am speaking now of opening into the cavity from the buccal side of the jaw.) If the engine is employed a spear-shaped drill is first used for opening into the antrum ; for enlarging this opening the drill is followed by a large, flame-shaped burr, coarse cut. The margins of the opening should be smoothed off by finer cut burrs. I have often employed a trephine for opening into the sinus.

Polypi are spoken of in some works on surgery as being one of the pathological conditions met with in the antrum. Some believe that they never originate in the antrum, but in the nasal cavity, and from there find their way into the antrum through the osteum maxillaire. They must be very rare, as Garretson does not mention them except as having grown into the antrum from the nose, and I have found them but twice, and both of these cases in the past year. They should be removed either by desiccation, evulsion, abscission or galvanic cautery.

Cysts are sometimes found in the antrum. They may have their origin in unerupted teeth or in the root of a pulpless tooth, or they may spring from the wall of the sinus with no apparent cause present. In some instances they grow in such proportions as to cause a thinning of the walls of the cavity, bulging of the eye, or of the cheeks, or of the hard palate. If the cause be a diseased tooth, its removal and the destruction of the walls of the cyst will end it. Cysts from either cause may be destroyed by laying them open and curetting the surface, or by applying iodin to their inner wall, or both of these means may be necessary.

Syphilis of the antrum has never come under my observation. Garretson says: "This is a disease which it might be inferred would have affinity for such mucous lined surfaces as the antrum. Now, the author's may be a singular experience, but in contradistinction of many who have written on the subject he must say that, with the wide scope afforded by such a hospital as that of Blockley, (in which for over a year he gave the study of the venereal disease a very close attention) he was unable to find a single case of disease of the antrum which could with justice be attributed to the vice.'' If syphilis be found affecting the antrum, specific medication will be necessary before a cure can be obtained.

Tumors of the antrum are said to be usually malignant, or likely to become so. The sarcomatous variety is the most common and unless there is entire ablation of the maxilla with the tumor, and then in the early stage of the growth, it is useless to attempt its removal, as death must soon follow. Tumors of a different nature may originate elsewhere and by growth find their way into the antrum, but the above statement is verified by the most careful and experienced observers.

Transillumination in the examination of the maxillary sinus for diagnostic purposes is lauded by some surgeons. Others are not so enthusiastic in the result obtained from its employment. My experience in the use of the electric lamp for this purpose does not lead me to believe that we may at all times depend upon it, as I have examined cases where there was certainly no pathological condition existing, and the sinus did not show up as clear as it did in some other cases, and again, I have examined cases which I knew to be engorged and the light passed through it, indicating no abnormal condition. If the fluid in the cavity be clear or semi-clear, it does not seriously obstruct the passage of the light; if, on the other hand, it be a purulent fluid, or if there be inspissated pus or growths of certain character, then the light does not show through so plainly. For the purpose of transillumination a dark room is necessary, an electric light is placed in the mouth, and the mouth closed.

In order to discover if pus flows into the nose from the antrum, the nostril should be cleansed and dried, then the patient should be directed to incline the head to the opposite side from that on which the disease is suspected, and held in this position for a few minutes. The nostril should then be dilated with a suitable speculum and a bright light reflected from a head mirror into cavity, when, if the osteum maxillary be not closed, if pus is present in any considerable quantity in the sinus, it will have been discharged and found in the nasal cavity.

